# Green Phenolic Resins from Oil Palm Empty Fruit Bunch (EFB) Phenolated Lignin and Bio-Oil as Phenol Substitutes for Bonding Plywood

**DOI:** 10.3390/polym15051258

**Published:** 2023-03-01

**Authors:** Kah Yen Lim, Tengku Arisyah Tengku Yasim-Anuar, Nur Sharmila Sharip, Farhana Aziz Ujang, Hazwani Husin, Hidayah Ariffin, Paridah Md Tahir, Xinping Li, Seng Hua Lee, Mohd Termizi Yusof

**Affiliations:** 1 Department of Microbiology, Faculty of Biotechnology and Biomolecular Sciences, Universiti Putra Malaysia (UPM), Serdang 43400, Selangor, Malaysia; 2 Research and Development Department, Nextgreen Pulp & Paper Sdn Bhd, Taman Tun Dr Ismail, Kuala Lumpur 60000, Malaysia; 3 Department of Bioprocess Technology, Faculty of Biotechnology and Biomolecular Sciences, Universiti Putra Malaysia (UPM), Serdang 43400, Selangor, Malaysia; 4 Institute of Tropical Forestry and Forest Products (INTROP), Universiti Putra Malaysia (UPM), Serdang 43400, Selangor, Malaysia; 5 Department Forest Production, Faculty of Forestry, Universiti Putra Malaysia (UPM), Serdang 43400, Selangor, Malaysia; 6 College of Bioresources Chemical and Materials Engineering, Shaanxi University of Science and Technology (SUST), Wei Yang District, Xi’an 710021, China; 7 Department of Wood Industry, Faculty of Applied Sciences, Universiti Teknologi MARA Pahang Branch Campus Jengka, Bandar Tun Razak 26400, Pahang, Malaysia

**Keywords:** black liquor, phenolic resins, phenolated lignin, bio-oil, adhesive

## Abstract

Lignin is a natural biopolymer with a complex three-dimensional network and it is rich in phenol, making it a good candidate for the production of bio-based polyphenol material. This study attempts to characterize the properties of green phenol-formaldehyde (PF) resins produced through phenol substitution by the phenolated lignin (PL) and bio-oil (BO), extracted from oil palm empty fruit bunch black liquor. Mixtures of PF with varied substitution rates of PL and BO were prepared by heating a mixture of phenol–phenol substitute with 30 wt.% NaOH and 80% formaldehyde solution at 94 °C for 15 min. After that, the temperature was reduced to 80 °C before the remaining 20% formaldehyde solution was added. The reaction was carried out by heating the mixture to 94 °C once more, holding it for 25 min, and then rapidly lowering the temperature to 60 °C, to produce the PL−PF or BO−PF resins. The modified resins were then tested for pH, viscosity, solid content, FTIR, and TGA. Results revealed that the substitution of 5% PL into PF resins is enough to improve its physical properties. The PL−PF resin production process was also deemed environmentally beneficial, as it met 7 of the 8 Green Chemistry Principle evaluation criteria.

## 1. Introduction

The good bonding capability and great durability of phenol-formaldehyde (PF) resin enable its wide application in the wood panel industry, particularly for outdoor applications. This adhesive is created by polycondensing phenol and formaldehyde, which yields two potential adhesives: novolac-type resins, which are produced under acidic conditions by using a molar excess of phenol over formaldehyde, and resol-type resins, which are produced under basic conditions by using a molar excess of formaldehyde over phenol [1]. However, each of these procedures require an acid or base catalyst, a formaldehyde cross-linker, and phenol derived from petroleum, making them non-renewable and largely dependent on the price and supply of fossil fuels. As such, biomass-derived phenolic chemicals such as lignin and bio-oil have been employed in PF resins in the past decades [2]. In this regard, the use of black liquor (BL) from pulp and paper industries is of great interest due to the major constituent of lignin in its composition [3,4,5,6].

However, the utilization of lignin for such purpose may be limited by its structural complexity and poor chemical reactivity, broad chemical differences depending on the source, processability, and depolymerization reaction [7,8]. Lignin modification to overcome these issues has been highlighted in numerous studies by which phenolation was deemed as one of the effective modification methods that can increase the phenolic hydroxyl group, hence, better reactivity [4,9,10]. In our previous study, the phenolation of lignin derived from an empty fruit bunch black liquor (EFB-BL) revealed a 51.5% phenolic hydroxyl content increment, indicating that more active sites in lignin are available to react with formaldehyde in a resin.

Not only that, the modification of lignin through its conversion to bio-oil is another interesting option. According to Kim [11], numerous studies have been reported showing the efficiency of bio-oil conversion from lignin with high phenol contents as a precursor in PF resin synthesis. In fact, the chemical parameters of that PF resin are similar to those of petroleum-based PF resin, demonstrating that bio-oil from pyrolysis products can be used as a phenol substitute for resin synthesis [12]. Similarly, our findings revealed the increment of phenolic hydroxyl and brominable content by 16.5 and 1.3 folds, upon conversion of EFB-lignin from the BL to bio-oil.

Even though the application of both phenolated lignin (PL) and bio-oil (BO) from biomass lignin as phenol substitute PF resin production has been widely reported, the utilization of both PL and BO originated and modified from the EFB-BL of paper industries are of great interest. In contrast to other agricultural biomass commonly used in the pulp and paper industry, such as eucalyptus, aspen, pine, bamboo, bagasse, and hemp, research into using oil palm biomass for commercial pulp production has only recently begun. The potential uses of its byproducts, including its black liquor, for other purposes are still the subject of extensive research. Despite numerous reports on lignin’s potential use, structure, and modification, more research is needed to understand issues associated with the physical and chemical variability of lignin extracted from oil palm biomass black liquor, as different raw material sources and pulping processes may influence its physical and chemical properties [1]. This reason justifies greater attention to the studies related to lignin extracted from EFB-BL. Aside from providing an alternative to petroleum-based phenol, this approach may enable the utilization of EFB-BL from the mill for the generation of high value-added products, following the waste-to-wealth and circular bio-economy concept.

It is previously reported that the phenolation and bio-oil conversion of the EFB lignin extracted for BL exhibited higher phenolic hydroxyl content, and may subsequently impart higher reactivity for PF synthesis. Therefore, this study aims to characterize the properties of green PF resins produced through phenol substitution by the PL and BO with varied phenol substitution rates. The properties of the synthesized PL−PF and BO−PF resins was evaluated according to the Chinese National Standard (GB/T 14732-2017) requirements of resins for wood industry adhesives and the Green Chemistry Principle. The findings of this study are also expected to shed new light on the rarely discussed use of EFB-PL and EFB-BO for green phenolic resin production.

## 2. Materials and Methods

### 2.1. Material

Black liquor for lignin extraction, phenolation and bio-oil conversion was collected from the pulping process of empty fruit bunch (EFB), called Preconditioning Refiner Chemical-Recycle Bleached Mechanized Pulp (PRC-RBMP) that had been carried out at a pulp mill; namely Nextgreen Pulp & Paper Sdn. Bhd. (Green Technology Park, Pekan, Pahang, Malaysia). The phenol (≥99.5%), formaldehyde solution (37%) and sodium hydroxide (97%) were purchased from Tianjin Damao Chemical Reagent Factory (Tianjin, China) and used as received.

### 2.2. Preparation of the Resin Adhesives

The phenol-formaldehyde (PF) resin adhesive is prepared according to [13]. The experiment was conducted in a three-necked flask equipped with a thermometer and a cooling condenser, and placed in a water bath to maintain a constant temperature. The mole ratio of the phenol to formaldehyde was set at 1:2 in this study. Variation of phenol substitution rate was executed as shown in Table 1. Firstly, a total of 10 g of phenol–phenol substitute was mixed in the flask. Then, 30 wt.% NaOH solution was added, followed by 80% of the formaldehyde solution. The mixture was heated to 94 °C and held for 15 min. The temperature was subsequently dropped to 80 °C prior addition of the remaining 20% formaldehyde solution. The mixture was heated up to 94°C again and hold for 25 min to complete the reaction, and quickly cooled to 60 °C to yield the PF resin.

### 2.3. Characterization of Resin Adhesive

#### 2.3.1. The pH, Viscosity, and Solid Content of the Adhesives

The pH, viscosity and solid content of the adhesives were determined in accordance with the Chinese National Standard (GB/T 14074-2017) [14]. A rotational viscometer was used to measure the viscosity of the resin. An appropriate amount of sample was added to a container with a diameter of not less than 3 cm and a height of not less than 11 cm. The rotor was then immersed vertically into the center of the sample to reach the rotor mark. The viscosity of the sample was measured and recorded. Meanwhile, solid content was measured by gravimetric analysis, whereby a 1 to 1.5 g sample was placed in a clean crucible and weighed with an analytical balance. The crucible was then dried in an oven at 120 °C for 2 h and transferred to a desiccator for cooling. Afterward, the constant weight was measured and recorded.

#### 2.3.2. FTIR Analysis

The Fourier transform infrared spectroscopy (FT-IR) spectra of all LPF resins were obtained using a Fourier transform infrared (FTIR) spectrometer (specification model: Vertex70, Bruker, MA, USA) in a frequency range of 500–4000 cm^−1^.

#### 2.3.3. Thermogravimetric Analysis

The thermal and curing behavior of the resins was determined by a synchronous thermogravimetry-differential scanning calorimetry (TG-DSC), thermal analyzer, specification model: STA449F3-1053-M. About 10 mg of the sample was weighed and placed on a balance in a furnace, and heat was applied over the temperature range from 30 °C to 800 °C. The experiments were performed in a dry nitrogen atmosphere and heated at a heating rate of 10 °C min^−1^.

### 2.4. Bonding Performance Evaluation of Plywood

Rubberwood veneers were used in the production of 3-layer plywood. Rubberwood veneers with dimensions of 1000 mm × 1000 mm × 4–5 mm (w × l × t) were obtained from Plus Intervest Sdn Bhd, Batu Kikir, Negeri Sembilan. The veneers were cut into 350 mm × 350 mm × 4–5 mm prior to plywood fabrication. Phenolated lignin phenol formaldehyde (PLPF) and bio-oil phenol formaldehyde (BOPF) resins were used to bond the rubberwood veneers after analysis and selection. Amounts of 5% and 10% phenolated lignin were used in the production of PLPF, and were denoted as 5PLPF and 10PLPF. Amounts of 5% and 10 % bio-oils were used in the production of BOPF, and were denoted as 5BOPF and 10BOPF. Phenol formaldehyde (PF) resin obtained from Aica Malaysia Sdn. Bhd., a local glue manufacturer located at Senawang, Selangor, served as control.

All PF resins were applied on one of the surfaces (single glueline) of rubberwood veneers at a glue spread rate of 200 g/m^2^. The veneers were then assembled into 3 layers perpendicularly. A cold press was applied to the assembled veneers for 10 min before being subjected to hot pressing at 140 °C for 10 min. The final thickness of the plywood was targeted at 12 mm by applying a stopper bar of 12 mm during pressing. Three plywood was produced for every type of PF resin.

The properties evaluation of plywood was done by bonding shear test. The pressed plywood was conditioned at 20 ± 2 °C and 65 ± 5% relative humidity until a constant mass was achieved. Ten test samples with dimensions of 25 mm × 50 mm × 12 mm were cut according to ISO 12466-1:2007. Prior to the bonding shear test, a series of boiling–drying–boiling pre-treatments were carried out.

The series of pre-treatments applied on the sample prior to testing were as follows. The samples for the bonding shear test were immersed for 24 h in water at 20 ± 3 °C. The samples were immersed for 6 h in boiling water and dried at room temperature for 1 h. The samples were then boiled for 4 h followed by cooling in water at <30 °C for at least 1 h. The samples for vacuum pressure were immersed in water and a vacuum of 85 kPa was applied for 30 min and followed by an immediate application of a pressure of 450 to 480 kPa for 30 min.

After pre-treatment, the dimension of each test samples was measured prior to bonding shear test according to ISO 12466-2:2007. The test was conducted by using Shimadzu Universal Testing Machine at a rate of 7 mm/min for 30 ± 10 s until the sample failed. The percentage of wood failure was also evaluated.

## 3. Results and Discussion

### 3.1. The Effect of Phenol Substitution Rate on pH, Viscosity, and Solid Content

The pH, viscosity and solid content are among the key indexes for adhesive [15]. As shown in Table 2, the properties of synthesised PL−PF and BO−PF were compared with Chinese National Standard (GB/T 14732-2017) requirements of resins for wood industry adhesives. All samples exhibited higher values compared to the standard requirement indicating a potential pathway for converting the phenolated lignin and bio-oil from oil palm biomass into viable adhesives.

In general, the influence of phenol substitution by EFB PL and BO from 5 to 30% substitution rate imparted no significant effect on the pH values. A negligible increment was noted by increasing the substitution rate for both EFB PL and BO. A similar finding was reported by Aslan et al. [16], who conducted a study on phenol substitution by phenol-rich fraction (PRF) of crude scotch pine wood bio-oil, whereby variation of the substitution rate up to 40% led to very minimal changes in pH (10.1 to 10.8). A study by Mao et al. [2], however, showed that increasing bio-oil for phenol substitution from 4 to 38% imparted a decrement in the pH values from 11.7 to 9.5, due to the presence of organic carboxylic acids in the bio-oil. Nevertheless, they also reported that neutralization of these carboxylic acids by the alkali used at the beginning of the resin synthesis could occur. In this study, the uses of NaOH may lead to the neutralization of acids in both PL and BO, hence, explaining the constant pH recorded.

**Figure 1:**
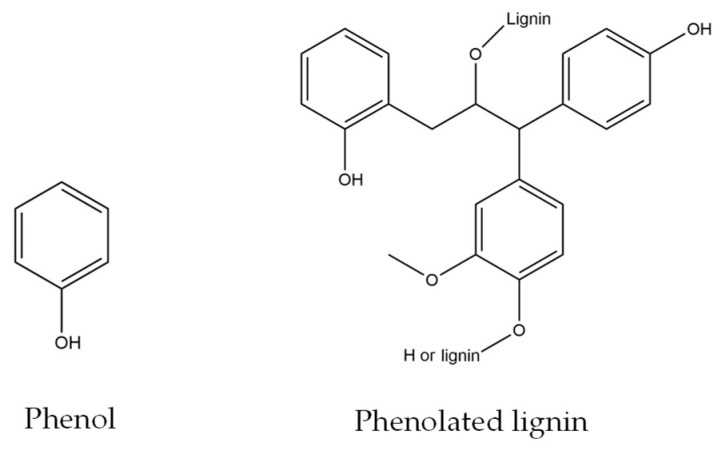
The chemical structure of phenol and phenolated lignin. (Figures redrawn from [17]).

Meanwhile, an increase in viscosity was observed with an increase in PL as a phenol substitute. The lowest substitution rate which is in 5PL−PF also exhibited higher viscosity than the non-substituted phenol PF sample. This is due to the high molecular weight of PL molecules, especially when compared to phenol, which has a more complicated structure than phenolated lignin (C_6_H_6_O) (Figure 1). A similar finding was reported by Yang et al. [13] who studied lignin–phenol–formaldehyde resin adhesives prepared with biorefinery technical lignin. Furthermore, this theory aligns with a report from [16] that states that, in addition to the high molecular weight properties, the dragging action of the phenol replacements molecules’ side chains may impact the rise in viscosity.

**Figure 2:**
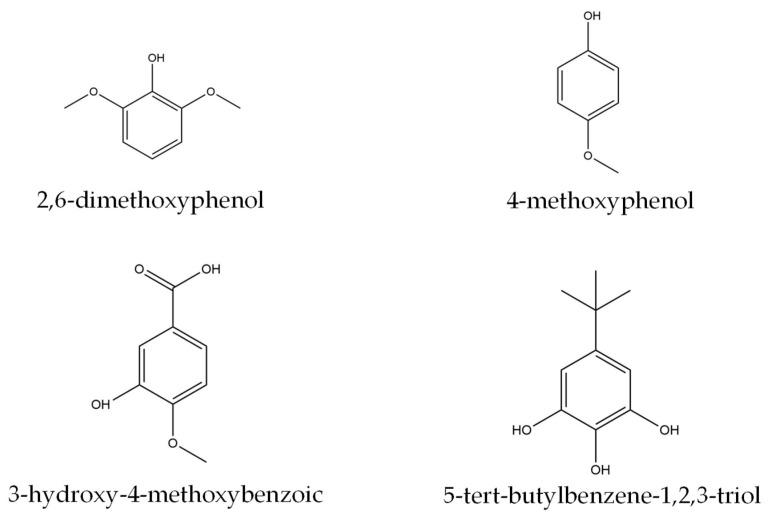
The chemical structure of several phenolic compounds in the BO.

In contrast to the PL−PF viscosity, the BO−PF exhibited much lower viscosity, and the values steadily decreased with an increased substitution rate. Despite composing varied phenolics compounds with higher molecular weight than phenol and side chains such as 2,6-dimethoxyphenol (C_8_H_10_O_3_), 4-methoxyphenol (C_7_H_8_O_2_), 3-hydroxy-4-methoxybenzoic acid (C_8_H_8_O_4_), and 5-tert-butylbenzene-1,2,3-triol (C_10_H_14_O_3_) (Figure 2), a dilution by the liquid BO in the presence of low boiling point organic compounds is expected [18,19]. This is in agreement with the findings of Ayrilmis and Özbay (2020) [20], who recorded lower viscosity when phenol was replaced with bio-oil made from Scots pine sawdust.

According to Podschun et al. [21], resin with high viscosity is preferred for its good performance as an adhesive. Nevertheless, it is important to note that the viscosity of resins in industrial applications may be varied depending on the purpose and applicator used. Cognard (2005) [22] highlighted the importance of adhesives viscosity adaptation to the application system, by which very fluid adhesive is needed for a spray gun and needle applicators. A too viscous adhesive may hinder the flow of adhesives through the spray nozzle or needle. On the other hand, extrusion guns need highly viscous adhesive, in order to resist sagging and flowing out of the joint. In terms of the application itself, Kamarudin et al. [23] regarded the unsuitability of too-thin resin to be used as a laminating material. Then, the results presented indicate the diversity of resin viscosity achieved by replacing petroleum-based phenols with PF and BO, indicating the advantages of these resources as greener and natural-based chemicals for adhesive synthesis.

Meanwhile, all PL−PF adhesives exhibited similar solid content to the PF except for the 30% substitution rate (30PL−PF) which was 56.51% (Table 2). Increased phenol substitution rate by PL gave no consistent trend and negligible changes to the solid content of the synthesised resin, by which the values are comparable to other works using lignin or black liquor for phenol substitution [13,24,25,26]. As for the BO−PF, decreasing solid content was observed with the increased phenol. This correlates with the viscosity and the aforementioned discussion whereby the dilution effect was responsible for this decreasing trend, which is in agreement with a published study [18].

### 3.2. Characterization of the PL−PF and BO−PF Resins

#### 3.2.1. UV Analysis

**Figure 3:**
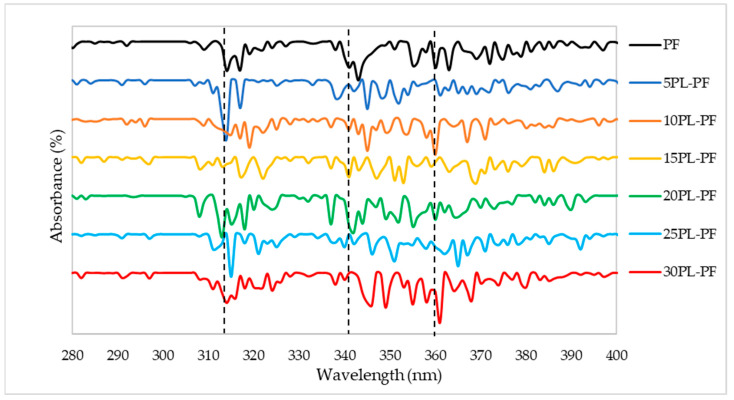
UV spectrum of the PL−PF resins from 5 to 30% phenol substitution rate.

**Figure 4:**
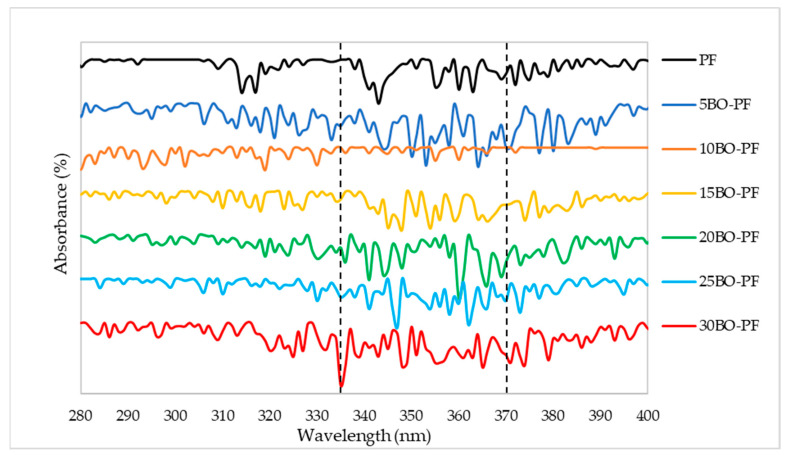
UV spectrum of the BO−PF resins from 5 to 30% phenol substitution rate.

The results presented in Figure 3 and Figure 4 are the UV characterisation of phenol–formaldehyde (PF) adhesive made by substituting PF with 5–30% PL and BO, respectively. This analysis was carried out to look for alterations in conjugated phenolics and the major phenolic groups contained in the PL−PF and BO−PF resins. Maximum absorption peaks were observed to appear around 315 and 345–360 nm on PL−PF as shown in Figure 3**,** and at around 335–375 nm on BO−PF, as shown in Figure 4. These maximum absorptions may be attributed to conjugated phenolic structures and a similar kind of result was also reported by Ramakoti et al. [27]. It was also observed that the PL−PF resins had a stronger absorption band between 320 to 350 nm than BO−PF, which could be due to phenolic hydroxyl group ionisation during phenolation. This suggests that specific and efficient phenolation at reactive sites in the side chains causes conjugated structures to disintegrate, resulting in an increase in phenolic hydroxyl content in PL−PF resins [28]. This also explains why PL−PF has a higher viscosity than BO−PF, as discussed in the preceding section.

Meanwhile, an increase in viscosity was observed with PL as a phenol substitute. The lowest substitution rate which is in 5PL−PF also exhibited higher viscosity than the non-substituted phenol PF sample. This is due to the high molecular weight of PL molecules, especially when compared to phenol, which has a more complicated structure than phenolated lignin. Overall, from this UV analysis, all PL−PF and BO−PF resins exhibited similar spectrums of UV, indicating that substitution of PL or BO does not have an effect on the structure of PF resins.

#### 3.2.2. FTIR Analysis

**Figure 5:**
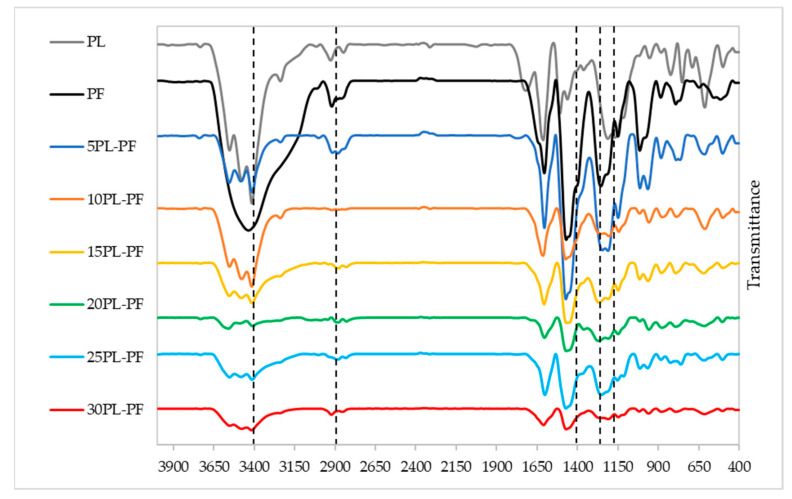
FTIR spectrum of the PL−PF resins from 5 to 30% phenol substitution rate.

The chemical structure of PF resin substituted with different substitution rates of PL and BO was investigated using FTIR analysis. Figure 5 shows the FTIR spectrum of PL−PF resins, and it can be observed that the absorption peak positions of the infrared spectrum of PL−PF resin at different substitution rates are generally comparable with the neat PF, with only the intensity varying slightly. This indicates a high degree of resemblance in terms of chemical nature of PF and PL−PF resins. Major differences in peak intensity were observed at around 1200–1290, 1450–1500, 2900–2920, and 3400–3500 cm^−1^.

According to Lai and Idris [29] and Talabi et al. [30], peaks at around 1211–1245 cm^−1^ indicate the presence of guaiacyl (G) propane units, whereas 1325–1375 cm^−1^ indicates the presence of syringyl (S) propane units. From the FTIR spectrum of PL−PF resins, it was discovered that all PL−PF resins have intense peaks around 1211–1245 cm^−1^ and no peak at 1325–1375 cm^−1^, indicating that all PL−PF resins are rich with G-propane units, thus making them suitable for phenolic resin production.

It was also observed that when phenol is substituted by PL, the peak intensity associated with the phenol-O characteristic band, which is at about 1200–1290 cm^−1^, becomes less intense. The trend shows that the higher the PL content, the lower the amount of phenol-O in the PL−PF resins, as it has been replaced by PL. The less phenol-O intensity then results in more PL branches in the PL−PF resins, which increases the viscosity of the resin. This then explains the trend of increasing viscosity of PL−PF resins when higher PL rates are incorporated in PF.

Similarly, the absorption band at around 1450–1500 cm^−1^ which corresponds to the presence of phenolic ring reduced when substituting some phenol with PL. In fact, after increasing the PL substitution rate, signal reduction continued to decrease at the same peak. The findings imply that phenol ring-containing molecules are higher in PF than PL−PF resins because PL does not solely contain phenol hydroxyl structure but also other side chains such as aldehydes, methyl, and aliphatic [9].

Meanwhile, peak intensities around 2900–2920 cm^−1^, which corresponds to the stretching vibration of C-H, -CH_2_ and -CH_3_, and peaks around 3400–3600 cm^−1^ which is attributed to the characteristic peak of aliphatic and aromatic OH groups or phenolic compounds, which steadily grow when more PL is substituted into the PF. This is because when PL is involved in the synthesis of PF resin, it carries its own group in the resin’s molecular chain, resulting in an increase in the number of functional groups in PL−PF resins [31]. Similar findings were also reported by [31,32,33,34], that studied the effect of substituting phenol with lignins for PF resin production. The authors discovered that PF resins substituting with lignins have more intense peaks of CH, OH groups and phenolic compounds in comparison to the neat PF resin.

**Figure 6:**
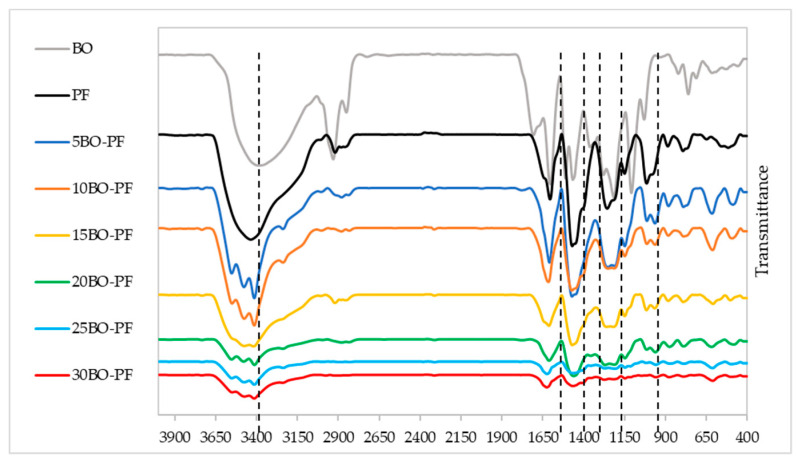
FTIR spectrum of the BO−PF resins from 5 to 30% substitution rate.

Meanwhile, Figure 6 shows the FTIR spectrum of BO−PF resins, and similar to PL−PF resins, the absorption peak positions of BO−PF resin at different substitution rates are comparable with the neat PF, proving that the BO−PF resins have a similar molecular structure to the neat PF. Nevertheless, the peak intensity varied slightly, especially at around 1030, 1211–1245, 1450–1460, 1600–1625 and 3200–3600 cm^−1^.

In contrast to PL−PF resins, an intense peak at around 1030 cm^−1^ was observed in BO−PF resins. This peak matches with C‒O bonds stretching vibration caused by alcoholic ether groups which may correspond to the aliphatic C–O(Ar), aliphatic C–OH, and methylol C–OH in the syringyl unit of lignin. Peaks at around 1211–1245 cm^−1^ which indicates the presence of G-propane units were also observed in BO−PF resins, but they are less intense compared to the PL−PF resins profiles.

The peak at approximately 1450–1460 cm^−1^, which is associated with methylene bridge was also observed in the spectra of PF and the peak becomes slightly intense when BO is gradually added to the PF. Similar to PL−PF resins, the peak intensity at around 1600–1625, and 3400–3600 cm^−1^ which was attributed to the aromatic ring vibrations, and OH groups, respectively, was also observed in BO−PF resins, suggesting the existence of the phenolic compounds in the resins [32].

To summarise, the peaks of BO−PF resins related to the above functional groups were remarkable, and from the FTIR spectra, it can be assumed that the BO, which has plenty of active ingredients such as phenols, aldehydes, ketones, and hydrocarbons can react with PF resin. Nonetheless, when a large amount of BO was added to the PF resin, some peaks became weaker, implying that adding too much BO would have a negative impact on the PF resin’s inherent molecular structure [18]. Indirectly, this would interfere with the formation of the inherent crosslinking structure and reduce the resin’s performance.

Based on the overall observation, both PL−PF and BO−PF resins show similar absorption peak positions as the neat PF, implying a chemical structural similarity before and after the substitution of PL and BO. It was also discovered that as the substitution rate of PL and BO increased, the intensity of several peaks around 1450–1625, 2900–2920, and 3200–3600 cm^−1^ became stronger, indicating a rise in phenolic OH content in the PL−PF and BO−PF resins. This FTIR result confirms that both PL and BO have been successfully introduced into the PF resin.

#### 3.2.3. Thermal Stability

**Figure 7:**
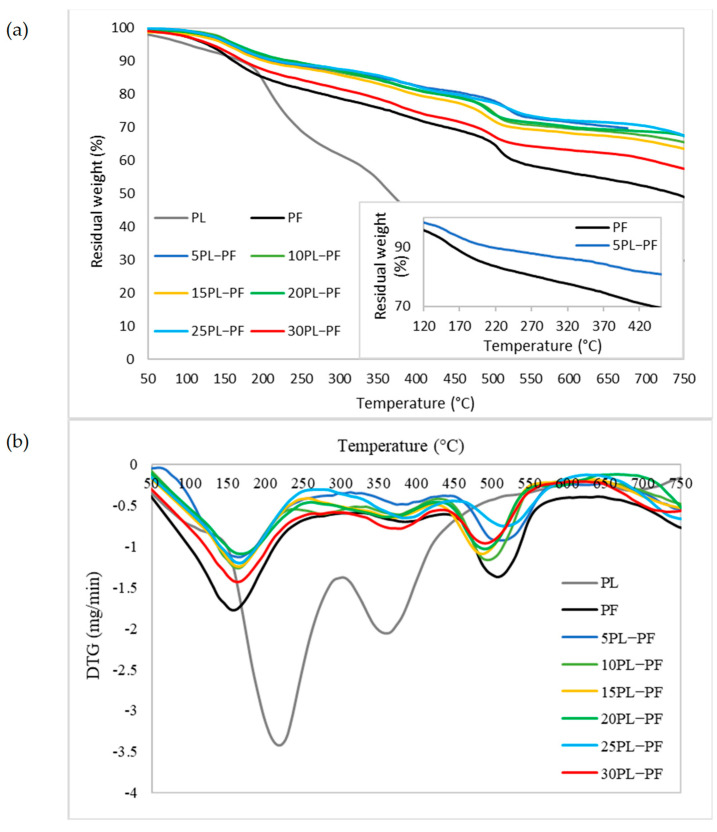
(**a**) TG and (**b**) DTG thermograms of the PL, PF and PL−PF resins with 5 to 30% phenol substitution rate, and the small box showing the TG thermogram of PF and 5PL−PF resins at 160 and 390 °C.

**Figure 8:**
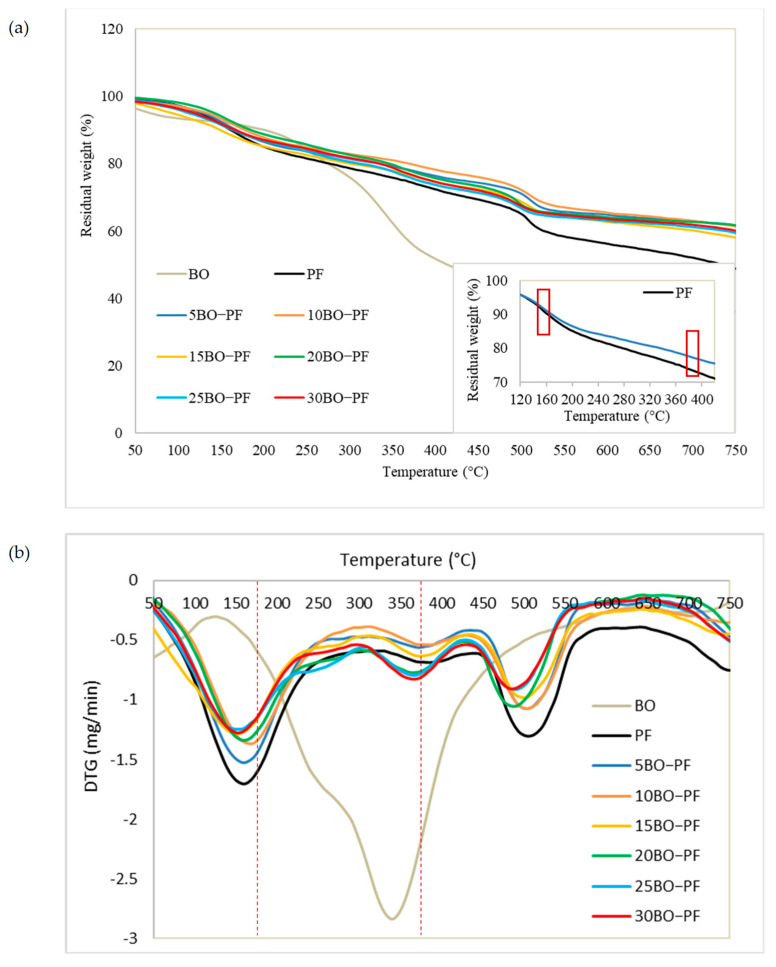
(**a**) TG and (**b**) DTG thermograms of the BO, PF and BO−PF resins with 5 to 30% phenol substitution rate, and the small box showing the TG thermogram of PF and 5BO−PF resins at 160 and 390 °C.

The thermogravimetric study was carried out to examine the thermal stability of PL−PF and BO−PF resins, and the TG and DTG thermograms shown in Figure 7 and Figure 8 demonstrate a broad temperature range at which PF, PL−PF, and BO−PF breakdown. This is typically caused by a number of oxygen-containing functional groups that are present and breaking down at varying temperatures [35]. An initial deterioration stage can be noticed at approximately 20 to 200 °C for both PL−PF and BO−PF resins. The evaporation of water in the phenolic gum, as well as the evaporation of free phenols and aldehydes, are the main causes of weight loss at this stage [9]. It was also discovered that the neat PF degraded faster than the ones with PL or BO at this early stage. The T_d10%_ of neat PF was observed to occur at around 157 °C, whereas the range of PL−PF and BO−PF resins was around 180–240 °C and 158–180 °C, respectively.

Notably, the degradation process could be delayed by increasing the amount of PL or BO in PF. For instance, the temperature of 10PL−PF after 10% degradation (T_d10%_) was roughly 225 °C, occurring 68 and 22 °C later than the neat PF (157 °C) and 5PL−PF (203 °C), respectively. However, when 15–30PL were added into PF, the temperature fell dramatically in a range of 171–239 °C, but still higher that the neat PF. A similar trend was also observed for BO−PF after 10% degradation, demonstrating BO’s ability to improve resin thermal stability. T_d10%_ was observed to occur at 165 and 173 °C after 5BO and 10BO were added into the PF resin, respectively, and the temperature dropped dramatically in a range of 159–180 °C after 15–30BO were added into PF. This reflected the result of FTIR which shows that PF has a higher composition of phenol and other side-chains such as aldehydes, methyl and aliphatic compared to the PL−PF and BO−PF resins, thus making it unstable and degraded faster than those modified ones. This result was also synchronised with the residual weight data. Theoretically, besides referring to the degradation temperature, an early degradation of a sample is also indicated by a high residual weight at a given temperature [32,36]. The TG thermograms demonstrate that the residual weight of PF at about 160 °C was around 90%, whereas PL−PF resin was in a range of 92–95% and BO−PF was in a range of 90–92% (Table 3). This result shows that 10% of PF resin has been degraded, while only 5–8% and 8–10% of PL−PF and BO−PF resins, respectively, have been degraded. This clearly shows that both PL−PF and BO−PF resins are more thermally stable than PF.

A similar degradation pattern was also observed in the second stage of degradation (T_d30%_) which occurred between 200 and 500 °C. This second degradation occurs mostly because of the breakage of ether and carbon–carbon bonds (inter-unit linkages) between different lignin units [24]. The TG thermogram shows that all PL−PF resins degraded later than the neat PF which degraded at 429 °C with 5PL−PF was found to be the most thermally stable as it degraded (T_d30%_) at 645 °C. This is probably because of the high H-OH and G-OH proportions of the PL−PF resins. Furthermore, unstable components such as phenol and aldehydes have been substituted with PL, and this helps in delaying the degradation process. Similar to T_d10%_, it was also observed that the incorporation of higher PL composition (10–30PL) in the PF was found to reduce the thermal stability as those resins degraded at around 473–587 °C (T_d30%_). To evaluate the temperature degradation trend with residual percentage, the residual percentage of each resin was recorded at 390 °C, where the second DTG peak of PF occurred. It was noticeable that by increasing a higher substitution rate of PL into PF, a higher residual weight was recorded, thus showing how stable the PL−PF resin is in contrast to the neat PF. At this temperature, all PL−PF resins had 3–14% higher residual weight than neat PF, with 5PL−PF resin having the highest residual weight at 82.9%, compared to 72.7% for neat PF.

Similar to PL−PF resin, all BO−PF resins were found to be more thermally stable than the neat PF at T_d30%_, as all of them degraded above 429 °C, with a delay of 45–82 °C. Among all BO−PF resins, 10BO−PF was found to be the most thermally stable as it degraded later than others at 511 °C. In terms of residual weight, BO−PF resins had 2.8–7.9% higher residual weight than neat PF, with 10BO−PF resin having the highest residual weight (78.5%) at this second degradation stage. Nevertheless, in contrast to PL−PF resins, which had residual weights between 75.2 and 82.9%, the residual weight of BO−PF resins is substantially lower, falling between 74.9 and 78.5%. This could be a result of the weakly bound propanoid components, such as methyl-, ethyl-, and vinyl-compounds, which are widely abundant in the BO−PF as depicted from the FTIR spectra (Figure 6) and can gradually degrade, resulting in a reduced percentage of residual weight [35]. Meanwhile, the high residual weight of PL−PF resins might be due to the higher content of aromatic structures in PL−PF which was contributed by PL after phenolation treatment. As a result, PL−PF resins are inherently more thermally stable than neat PF and BO−PF resins.

The TG thermograms also revealed that at the final stage of degradation, which was around 750 °C, the residual weight of PL−PF resins was higher than both neat PF and BO−PF, which varied from 57.6 to 66.8%, with the maximum residual weight obtained by 5PL−PF. Meanwhile, BO−PF resins recorded a residual weight of around 58–61.7%, which was 18.9–26.2% higher compared to the neat PF. This shows that the substitution of PL and BO into the PF can improve PF resin’s thermal stability.

In summary, the thermogravimetric analysis clearly shows that the thermal degradation and stability of resins are different due to the chemical and physical modification of their structure. More importantly, the TGA results show that 5PL−PF resin is most thermally stable due to its fullness of aromatic rings and a high proportion of H-OH and G-OH, as depicted from the FTIR spectrum compared to both neat PF and BO−PF.

### 3.3. Performance Evaluation

Overall physical analyses showed that 5PL−PF and 5BO−PF have greater physical properties as compared to other formulations. The performance of 5PL−PF and 5BO−PF as modified resins was, therefore, evaluated in order to determine which modified resin is the best. The performance evaluation was carried out by scoring the resins’ performance into two categories; physical properties and sustainable production.

In the context of physical properties, 5PL−PF and 5BO−PF possess better characteristics than the neat PF. Both 5PL−PF and 5BO−PF exhibit similar pH values to the neat PF, and the same trend was seen for viscosity and solid content. However, when comparing 5PL−PF and 5BO−PF, 5PL−PF has a greater viscosity and solid content, at 131.4% and 19.1%, respectively. According to Wahab et al. [37], a higher viscosity will result in quicker gelation of the resin. This demonstrates that 5PL−PF, as compared to 5BO−PF, can gel in a shorter amount of time. Additionally, a thermal stability analysis showed that 5PL−PF is more thermally stable than both 5BO−PF and neat PF, making it far more practical to utilise on a variety of materials.

Meanwhile, in the context of sustainable production, the green chemistry principle was used as a guideline to evaluate both 5PL−PF and 5BO−PF. Green chemistry’s twelve principles are design criteria or guidelines that serve as a foundation for developing ecologically beneficial and long-lasting goods [38]. Among the twelve principles of green chemistry, 6 criteria were chosen based on the Multiple Criteria Decision Aiding (MCDA) model to evaluate the 5PL−PF and 5BO−PF production [39]. Those criteria are prevention, use of less hazardous chemicals, energy efficiency, use of renewable feedstock, process effectiveness and energy consumption. Table 4 shows a summary of the evaluation.

The overall sustainability evaluation revealed that both 5PL−PF and BO−PF fulfilled 5 out of 6 criteria of the green chemistry principle. Both 5PL−PF and 5BO−PF were awarded a score for the 1st (prevention) and 4th (use of renewable feedstock) criteria because both are made from BL, which is a renewable by-product of the pulping process. These criteria aims to avoid the creation of waste by using it for the creation of value-added products. A score was also given to both modified resins on 2nd criteria, as no hazardous chemicals were used during phenolation or microwave pyrolysis processes. Both production methods do not cause toxicity to humans and environment.

Nevertheless, only 5BO−PF was given a score on energy efficiency (3rd criteria) and process effectiveness (5th criteria), as it only takes 15 min of microwave pyrolysis to produce 5BO−PF, whereas, 110 min of phenolation process is required to produce 5PL−PF. The use of 5BO−PF was seen to be more energy efficient as it was produced at higher productivity, with a difference of 95 min with 5PL−PF. Nevertheless, despite the short 5BO−PF production time, BL initially needs to be exposed to very high temperatures and microwave radiation, and this requires a lot of energy, in turn affecting its energy consumption score. In this regard, 5PL−PF received a score because BL doesn’t have to be exposed to extremely high temperatures or significant pressure during the phenolation process.

It is notable that both 5PL−PF and 5BO−PF received 5 points in the context of the green chemistry concept. However, when physical properties were added together, 5PL−PF outperformed 5BO−PF and received a final score of 7 out of 8. Thus, 5PL−PF can be considered as an effective, safe and environmentally friendly resin material.

### 3.4. Bonding Performance of Plywood

The shear strength and wood failure of 3-ply rubberwood plywood bonded with commercial (PF), 5PLPF, 10PLPF, 5BOPF, 10BOPF and 10LPF (PF with 10% unmodified lignin substitution) resins are shown in Table 5.

Under a dry condition, all plywood recorded bonding shear higher than 1.0 MPa, meeting the minimum requirement of standard ISO 12466-2:2007. The estimated wood failure percentage ranges between 68.5% to 90.6%, which suggests that plywood has satisfactory bonding performance. It should be noted that plywood bonded with commercial PF resin (control) has a significantly higher shear strength of 2.72 MPa compared to that of PLPF-, BOPF- and LPF-bonded plywood. Meanwhile, plywood bonded with phenolated lignin (1.61 to 1.78 MPa) and bio oil (1.24 to 1.58 MPa) has better shear strength than plywood bonded with unmodified lignin (1.05 MPa). Phenolated lignin was reported to be able to improve the mechanical properties of raw lignin in phenolic because phenolation improved lignin reactivity by increasing the number of reactive sites for crosslinking to occur. The findings in this study suggested that replacement of phenol with 5% phenolated lignin is sufficient to yield a satisfactory result. Podschun et al. [4] reported that an improvement of potential crosslinking sites by a factor of 12.9 for phenol formaldehyde resins was obtained after phenolation process of raw lignin. In addition, phenolated lignin also showed higher solubility than raw lignin. However, the reason why PLPF resin performed inferiorly compared to pure PF resin remains uncertain. Podschun et al. [21] speculated that the occurrence of de-phenolation during resin synthesis might be a probable reason for such observation. Similar observations were obtained for shear strength under wet conditions, boil-dry-boil and vacuum pressure.

On the other hand, studies reported that the replacement of phenol with bio-oil yielded plywood with stronger bonding than the plywood bonded with commercial PF resin [40,41]. Mohamad Hafiz [42] found that replacing 20% of the phenol with tannin produced a tannin phenol–formaldehyde (TPF) with performance comparable to that of neat PF. One of the reasons could be that bio-oil contains a high amount of hydroxyl methyl and hydrocarbons that aid to form the crosslinking structure with the wood veneers [41]. However, in this study, plywood bonded with 5% and 10% bio oil did not possess better bonding performance than the control plywood. Nevertheless, the produced plywood is still able to achieve the minimum requirement stipulated in the standard.

The results indicate that the plywood bonded with PLPF and BOPF resin is appropriate for veneer plywood intended for exposure to weather over sustained periods (Class 3), according to the requirements specified in ISO 12466-2:2007.

## 4. Conclusions

Both PL and BO were found to be successful in improving the thermal properties of PF resin, as well as being much less toxic and more environmentally friendly than the currently available petroleum-based PF resin. This also demonstrates the applicability of lignin extracted from BL for the production of bio-based resin, which opens up BL’s potential as a feedstock for the development of value-added products. Despite the fact that their properties are comparable, the overall performance evaluation, however, demonstrates that 5PL−PF resin is more effective than all BO−PF, particularly in terms of physical properties and sustainable processing.

## Figures and Tables

**Table 1:** The variation of phenol substitute and the substitution rate.

Adhesive	Phenol Substitution Rate (%)	Phenol (g)	Phenol Substitute (g)	
			EFB Phenolated Lignin (PL)	EFB Bio−Oil (BO)
PF	0	10	0	-
5PL−PF	5	9.5	0.5	-
15PL−PF	15	8.5	1.5	-
30PL−PF	30	7	3	-
5BO−PF	5	9.5	-	0.5
15BO−PF	15	8.5	-	1.5
30BO−PF	30	7	-	3

**Table 2:** The adhesive properties in comparison to the Chinese National Standard (GB/T 14732-2017).

Adhesive	pH	Viscosity (cP)	Solid Content (%)
GB/T14732-2017	≥7.0	≥60.0	≥35.0
PF	10.31	235	51.62
5PL−PF	10.67	324	51.91
10PL−PF	11.00	466	50.51
15PL−PF	11.12	1169	50.22
20PL−PF	11.56	1700	50.91
25PL−PF	11.39	2172	52.14
30PL−PF	11.00	3608	56.51
5BO−PF	10.92	140	43.57
10BO−PF	10.98	128	34.78
15BO−PF	10.91	127	48.47
20BO−PF	11.13	106	40.04
25BO−PF	11.13	94	34.60
30BO−PF	11.20	99	37.61

**Table 3:** Residual weight of PL, BO, PF, PL−PF resins and BO−PF resins.

	Residual Weight of PL−PF Resins (%)				Residual Weight of BO−PF Resins (%)		
	160 °C	390 °C	750 °C		160 °C	390 °C	750 °C
PL	91.0	44.6	29.8	BO	92.0	52.4	36.2
PF	90.2	72.7	82.8	PF	90.2	72.7	48.8
5PL−PF	94.3	82.8	66.8	5BO−PF	91.0	76.7	61.6
10PL−PF	95.4	81.6	65.4	10BO−PF	92.1	78.5	61.5
15PL−PF	94.0	80.3	63.6	15BO−PF	88.6	74.9	58.0
20PL−PF	95.4	81.6	67.5	20BO−PF	92.7	76.0	61.7
25PL−PF	94.8	82.8	67.5	25BO−PF	90.5	74.1	59.4
30PL−PF	91.9	75.2	57.6	30BO−PF	90.7	75.0	60.2

**Table 4:** Evaluation of the production of PL−PF and BO−PF resins based on the physical properties and green chemistry principle.

Criteria	5PL−PF	5BO−PF
	Properties	
Physical properties	1	0
Thermal stability	1	0
	Green chemistry principle	
Prevention	1	1
Use of less hazardous chemical	1	1
Energy efficiency	0	1
Use of renewable feedstock	1	1
Process effectiveness	1	1
Energy consumption	1	0
Total Score	7/8	5/8

Note: 1: Positive impact; 0: Negative impact.

**Table 5:** Shear strength and wood failure of rubberwood plywood bonded with EFB phenolated lignin phenol formaldehyde (PLPF) and bio oil phenol formaldehyde (BOPF) resin.

Type	Dry	Soak 24 h	Boil-Dry-Boil	Vacuum Pressure	Wood Failure (%)
Control	2.72 ± 0.12	2.41 ± 0.05	1.17 ± 0.12	1.36 ± 0.06	90.6 ± 4.6
5PLPF	1.78 ± 0.10	1.56 ± 0.06	0.77 ± 0.13	1.14 ± 0.13	84.2 ± 2.3
10PLPF	1.61 ± 0.09	1.35 ± 0.07	0.75 ± 0.05	1.02 ± 0.07	83.0 ± 2.3
5BOPF	1.58 ± 0.19	1.38 ± 0.09	0.69 ± 0.09	1.09 ± 0.11	77.9 ± 3.8
10BOPF	1.24 ± 0.12	1.13 ± 0.08	0.55 ± 0.10	0.88 ± 0.04	70.5 ± 2.2
10LPF	1.05 ± 0.15	0.87 ± 0.10	0.57 ± 0.03	0.76 ± 0.08	68.5 ± 1.6

Note: Numbers after “±” is standard deviation.

## Data Availability

Not applicable.
